# A Bayesian Surprise Approach in Designing Cognitive Radar for Autonomous Driving

**DOI:** 10.3390/e24050672

**Published:** 2022-05-10

**Authors:** Yeganeh Zamiri-Jafarian, Konstantinos N. Plataniotis

**Affiliations:** Department of Electrical and Computer Engineering, University of Toronto, Toronto, ON M5S 3G4, Canada; kostas@ece.utoronto.ca

**Keywords:** cognitive radar, Bayesian surprise, expectation of Bayesian surprise, linear Gaussian dynamic systems

## Abstract

This article proposes the Bayesian surprise as the main methodology that drives the cognitive radar to estimate a target’s future state (i.e., velocity, distance) from noisy measurements and execute a decision to minimize the estimation error over time. The research aims to demonstrate whether the cognitive radar as an autonomous system can modify its internal model (i.e., waveform parameters) to gain consecutive informative measurements based on the Bayesian surprise. By assuming that the radar measurements are constructed from linear Gaussian state-space models, the paper applies Kalman filtering to perform state estimation for a simple vehicle-following scenario. According to the filter’s estimate, the sensor measures the contribution of prospective waveforms—which are available from the sensor profile library—to state estimation and selects the one that maximizes the expectation of Bayesian surprise. Numerous experiments examine the estimation performance of the proposed cognitive radar for single-target tracking in practical highway and urban driving environments. The robustness of the proposed method is compared to the state-of-the-art for various error measures. Results indicate that the Bayesian surprise outperforms its competitors with respect to the mean square relative error when one-step and multiple-step planning is considered.

## 1. Introduction

Despite a precipitous drop in driving during the pandemic, the Governors Highway Safety Association (GHSA) of the United States reported that 2020 had the most significant annual increase in pedestrian deaths [1]. This shocking report indicated that the fatality rate for pedestrians spiked by 21% compared to the previous year. Big technology companies have invested in making autonomous radar an integral part of safety systems to prevent such accidents [2].

Current driver assistance technologies use a combination of sensors (e.g., radar, LiDAR (light detection and ranging), camera, GPS, etc.) and software to identify certain safety risks that help the driver to avoid accidents [3,4]. Compared to video cameras and LiDAR, a radar sensor is unaffected by bad weather and light conditions, and it can also detect hidden targets behind other vehicles [5,6]. Undeniably, a well-designed radar system that further advances safety benefits is indispensable for the evolution of automotive technology.

Cognitive radar, first introduced by S. Haykin [7], is an engineering tool to build intelligent tracking sensors which will eventually make autonomous driving a reality [8]. The model was inspired by the perception–action process that takes place in the brain [9]. The cognitive radar continuously interacts with its surroundings to gather information and adapts its operating parameters to ensure accurate target tracking without human control. The sensor selects a transmit waveform that anticipates a better estimate of the target’s state (i.e., distance, velocity) based on the information provided by the received radar measurements. The challenge in designing cognitive radar arises from the uncertain environment [10]. The design objective is to achieve an estimate of the target’s state that minimizes the mean squared error and to gain informative radar measurements in the presence of such disturbances. To this end, this paper focuses on quantifying new information from noisy radar measurements to improve the state estimation process over time.

In the perception and learning literature, surprising events which violate prior expectations encourage information-seeking behaviors that affect learning and decision making [11]. Surprise is an emotion resulting from a discrepancy between an expectation and an actual observation [12]. It measures the amount of information that is associated with an unexpected event [13]. Several definitions and expressions of surprise have been proposed in previous studies [14,15,16,17,18]. The most common forms of surprise are the Shannon surprise [14], the Bayesian surprise [15], and the free energy [16]. For a biological agent, the Shannon surprise measures the unlikeliness of an outcome [14]. Meanwhile, the Bayesian surprise measures how much an agent’s expectation changes when a new observation is made [15]. In [16], the free energy principle suggests that biological agents make decisions by reducing the Shannon surprise, and adjust their (internal) models to make better predictions by minimizing the Bayesian surprise.

This research adopts surprise as the main methodology to measure the amount of new information within the received radar measurements. In particular, the paper considers the Bayesian surprise since it computes how much information a new measurement provides to estimate future states based on prior knowledge. In previous works, the Bayesian surprise has been applied to different models and applications to acquire information from data [13,19,20,21,22]. In [13,19], the Bayesian surprise measures attention and anticipates the human gaze to enhance computer vision applications. A similar attempt is followed in [20], where the Bayesian surprise detects anomalies for autonomous guided vehicles in an unsupervised fashion. In associative learning, the Bayesian surprise is also employed as an error-correction learning rule for the Rescorla–Wagner model [21]. A recent paper considers a Bayesian interpretation of surprise-based learning to perform model estimation [22]. It should be noted that selecting informative measurements using Bayesian surprise to improve state estimation can be viewed as active measurement selection for regression analysis. Similar ideas have been researched and implemented in the literature, and several previously shown results could be interpreted and re-introduced through the Bayesian surprise framework. The interested reader may wish to consult [23,24,25] for input and insight. Our theory is that the Bayesian surprise framework will help understand commonalities amongst methods, lead to interesting connections, and inspire future developments.

For a simple vehicle-following scenario, this article proposes a new design of cognitive radar that operates based on the Bayesian surprise. This research generates radar measurements from a family of linear Gaussian dynamic systems. Assuming that the parameters of the system are known, the Kalman filter [26] is applied as the optimal estimator in the mean squared error sense. Given the current estimated target’s state, the sensor plans by measuring how much information each waveform—available from a predefined set—contributes to state estimation, and selects the one that conveys the maximum information based on the Bayesian surprise. In addition, the paper investigates the estimation algorithms for one-step and multiple-step planning. This proposed design assumes that the sensor is equipped with a predefined set of measurement noise covariances, where each one corresponds to a distinct waveform. Compared to other forms of surprise [14,16], the authors of this paper anticipate that the Bayesian surprise provides sufficient information to minimize the state estimation error.

Despite significant achievements in designing cognitive radar [9,27,28,29], there remains limited literature that compares radar designs based on the choice of information measure and its associated waveform-/measurement-selection procedure. For the first time, this paper systemically analyzes the works in [16,27,30] as alternative methods for designing cognitive radar. Except in [27], where the Shannon entropy is used to quantify the information within radar measurements, the free energy principle [16] and the influence matrix [30] have not been directly addressed to solve the state estimation problem in cognitive radar. In addition, this work re-introduces these methods in the context of linear Gaussian dynamic models and shows how they are related to the proposed approach.

Numerous experiments are carried out to comprehensively evaluate and compare the estimation performance of the proposed cognitive radar with the state-of-the-art. The millimeter-wave radar sensor presumes transmitting frequency-modulated continuous wave (FMCW) signals operating in the 77 GHz frequency band [31]. The paper designs the parameters of the FMCW radar in a manner that supports single-target tracking in highway and urban environments. The paper considers various error measures to examine different aspects of estimation performance. The credibility of the proposed estimation algorithm is ranked based on a pairwise comparison scheme [32]. Simulation results determine whether the tracking performance is improved when the radar switches from one-step planning to multiple-step planning.

The rest of this paper is organized as follows. Section 2 presents the model assumptions and defines the research objective in designing cognitive radar for a simple vehicle-following scenario. Section 3 briefly reviews prior works and demonstrates our proposed method to solve the state estimation problem in cognitive radar. Section 4 evaluates the estimation performance of the proposed approach by emulating real-life driving scenarios. Results are compared to alternative designs for different error measures. Finally, Section 5 concludes the paper.

### Notation

In this paper, scalar variables are represented by non-bold lowercase letters (e.g., *c*), the vectors are denoted by bold lowercase letters (e.g., x), and matrices and sets of vectors are shown as uppercase bold letters, (e.g., F). In addition, tr{.}, |.|, and ||.|| represent the trace operator (e.g., tr{A}), the determinant operator (e.g., |A|), and the norm operator (e.g., ${\left||x|\right|}_{P^{-1}}^{2}=x^{T}P^{-1}x$), respectively. Moreover, ${\left\{.\right\}}^{T}$ applies the transpose operation on matrices (e.g., $F^{T}$).

## 2. Problem Formulation

**Figure 1:**
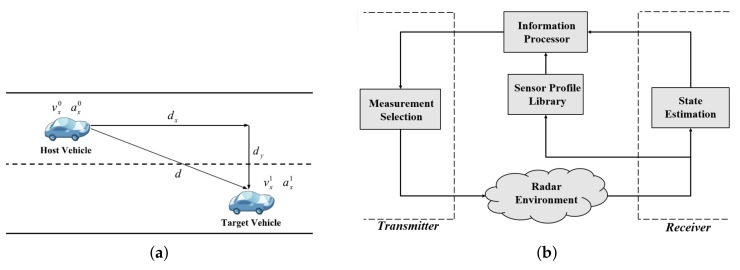
(**a**) A simple vehicle-following scenario [28] and (**b**) the block diagram of the cognitive radar as an autonomous system.

Figure 1a illustrates a simple vehicle-following scenario, where a cognitive radar is mounted on the host vehicle, tracking the state dynamics of a target vehicle (i.e., distance, velocity, etc.). Let us consider that something unexpected occurs, and the dynamics of the target vehicle change. To avoid a collision, the cognitive radar must be able to detect these changes to adjust the dynamics of the host vehicle accordingly. The radar signal received at the host vehicle provides information about the target’s state. According to this information, the cognitive radar makes a decision and sends a waveform (or signal) that can provide a better estimate of the target’s state at future time instances.

The goal of the cognitive radar is to ensure reliable and accurate tracking of the target over time. To accomplish this objective, Figure 1b presents a simple design of a cognitive radar as an autonomous system. Inspired by the cognitive dynamic system [27], the model consists of a radar environment, a receiver, an information processor, and a transmitter. The target of interest (i.e., target vehicle) is embedded in the radar environment. It is assumed that the system is equipped with a sensor profile library containing several types of waveforms. Let us consider the stages that the cognitive radar undergoes for a single cycle. Note that the term "cycle" refers to the processes that take place at one time instant. An estimate of a target’s state is determined at the receiver by processing measurements from the radar environment. The information processor measures how much information each waveform—available from the sensor profile library—contributes to estimating the target’s state for the next cycle by planning multiple time steps. Finally, the transmitter selects the waveform that leads to informative radar measurements and provides an improved estimate of the target’s state. The sensor applies the chosen waveform to the radar environment and repeats the same cycle.

This research proposes a holistic methodology to quantify information and maintain informative radar measurements that minimize the state estimation error over time. To this end, the following introduces the assumptions made to model the cognitive radar and formulates the design objectives of this research.

### 2.1. Model Assumptions

The following presents the model assumptions to construct radar measurements for the simple vehicle-following scenario and addresses the sensor profile library.

#### 2.1.1. Linear Gaussian Dynamic System

For the simple driving case depicted in Figure 1a, the radar measurements at time index *k*, denoted as $z_{k}\in R^{m}$, are obtained from a set of linear Gaussian state-space models [26], expressed as follows:

(1) $$\begin{array}{cc} x_{k+1} & =F_{k}x_{k}+w_{k}\\ z_{k} & =H_{k}x_{k}+v_{k} \end{array}$$

where the evolution of the state vector, denoted as $x_{k}\in R^{n}$, follows a first-order Markov chain process. In this problem, the state represents the entities of motion regarding the host and target vehicle, written as

(2) $$x_{k}={\left[\begin{array}{c} v_{x,k}^{0},a_{x,k}^{0},d_{x,k},v_{x,k}^{1},a_{x,k}^{1} \end{array}\right]}^{T}$$

where $v_{x,k}^{0}$ and $a_{x,k}^{0}$ are the velocity and acceleration of the host vehicle; $v_{x,k}^{1}$ and $a_{x,k}^{1}$ are the velocity and acceleration of the target vehicle; $d_{x,k}$ represents the longitude distance between the two cars. In Equation (1), $F_{k}\in R^{n\times n}$ and $H_{k}\in R^{m\times n}$ are, respectively, the transition matrix and the measurement matrix. Meanwhile, the state noise $w_{k}\in R^{n}$ and measurement noise $v_{k}\in R^{m}$ are assumed additive zero-mean white Gaussian processes, where $Q_{k}\in R^{n\times n}$ and $R_{k}\in R^{m\times m}$ are the state noise covariance and the measurement noise covariance, respectively. Note that the initial state follows a Gaussian distribution, denoted as $x_{0}∼N\left(\hat{x}\left(0|0\right),P\left(0|0\right)\right)$, and is mutually uncorrelated with the noise elements.

According to the equations of motion that presume constant acceleration, $F_{k}$ and $Q_{k}$ are derived as [33]

(3) $$\begin{array}{c} F_{k}=\left[\begin{array}{ccccc} 1 & T_{s} & 0 & 0 & 0\\ 0 & 1 & 0 & 0 & 0\\ -T_{s} & -T_{s}^{2}/2 & 1 & T_{s} & T_{s}^{2}/2\\ 0 & 0 & 0 & 1 & T_{s}\\ 0 & 0 & 0 & 0 & 1 \end{array}\right], \end{array}$$

and

(4) $$\begin{array}{c} Q_{k}=\left[\begin{array}{ccccc} T_{s}^{4}/4 & T_{s}^{3}/2 & -T_{s}^{5}/12 & 0 & 0\\ T_{s}^{3}/2 & T_{s}^{2} & -T_{s}^{4}/6 & 0 & 0\\ -T_{s}^{5}/12 & -T_{s}^{4}/6 & T_{s}^{6}/18 & T_{s}^{5}/12 & T_{s}^{4}/6\\ 0 & 0 & T_{s}^{5}/12 & T_{s}^{4}/4 & T_{s}^{3}/2\\ 0 & 0 & T_{s}^{4}/6 & T_{s}^{3}/2 & T_{s}^{2} \end{array}\right]\sigma_{q}^{2} \end{array}$$

where $T_{s}$ and $\sigma_{q}^{2}$ refer to the sample time and the state noise variance, respectively. Since the dynamics of the target vehicle is of interest, the measurement matrix is assigned as

(5) $$H_{k}=\left[\begin{array}{ccccc} 0 & 0 & 0 & 1 & 0\\ 0 & 0 & 1 & 0 & 0 \end{array}\right]$$

where the velocity of the target vehicle, $v_{x,k}^{1}$, and the longitude distance, $d_{x,k}$, are the available radar measurements. The choice of the measurement noise covariance depends on the waveform that the radar sensor conveys for target tracking. FMCW is the most well-known modulation format, where linear frequency ramps with different slopes are transmitted [6]. The FMCW modulation with a Gaussian-shaped pulse is commonly used in designing autonomous radars since it exhibits excellent range and velocity resolution. Thus, for a Gaussian-shaped pulse with FMCW modulation, $R_{k}$ is defined as follows [34]:

(6) $$R_{k}\left(\lambda_{k-1},b_{k-1}\right)=\left[\begin{array}{cc} \frac{c^{2}}{{\left(2\pi f_{c}\right)}^{2}\eta }\left(\frac{1}{2\lambda_{k-1}^{2}}+2\lambda_{k-1}^{2}b_{k-1}^{2}\right) & -\frac{c^{2}b_{k-1}\lambda_{k-1}^{2}}{2\pi f_{c}\eta }\\ -\frac{c^{2}b_{k-1}\lambda_{k-1}^{2}}{2\pi f_{c}\eta } & \frac{c^{2}\lambda_{k-1}^{2}}{2\eta } \end{array}\right]$$

where $\lambda_{k-1}$, $b_{k-1}$, *c*, $f_{c}$, *B*, and η are the pulse duration, the chirp rate, the speed of light, the carrier frequency, the signal bandwidth, and the received signal-to-noise ratio (SNR), respectively. As shown in Equation (6), the measurement noise covariance depends on the pulse duration and chirp rate at the k−1 time index. This indicates that the system’s selection of the transmitted waveform (i.e., $\lambda_{k-1}$ and $b_{k-1}$) at the previous time cycle influences the radar measurements (i.e., $z_{k}$) at the current cycle. Both $\lambda_{k-1}$ and $b_{k-1}$ are the design parameters that signify the radar waveform based on the tracking application (e.g., single- or multiple-target tracking). Since the transmitter and the receiver of the radar sensor are both positioned on the host vehicle, the received SNR for the target vehicle located at distance $d=\sqrt{d_{x}^{2}+d_{y}^{2}}$ may be obtained as [34]

(7) $$\eta ={\left(\frac{d_{0}}{d}\right)}^{4}$$

where $d_{y}$ is the lateral distance and $d_{0}$ is the distance at which 0 dB SNR is achieved.

Note that linear Gaussian dynamic systems suffice to model the motion dynamics when simple driving is assumed. However, modeling complex driving situations that consider multiple targets requires switching dynamic models that may not be necessarily expressed as in Equation (1).

#### 2.1.2. Sensor Profile Library

For the model illustrated in Figure 1b, the cognitive radar is assumed to be equipped with a prescribed set of measurement noise covariances, referred to as the sensor profile library. According to Equation (6), the measurement noise covariance is computed based on the waveform parameters: pulse duration and chirp rate. The sensor profile library holds a large set of measurement noise covariances, denoted as R. Since it is computationally expensive and time-consuming to go through the entire library at each time cycle to select the informative measurement (or optimum waveform), a localized set is adopted instead. As a solution, this paper considers a k-nearest neighbors (kNN) method to obtain the localized set $R_{k}^{L}=\left\{R^{\left(1\right)},R^{\left(2\right)},\cdots ,R^{\left(N_{L}\right)}\right\}\in R$, which includes measurement noise covariances that are neighbors to $R_{k}$. The work in [28] views this localization approach as a form of an attention mechanism, which is one of the basic principles of cognition in modeling intelligent radar sensors.

### 2.2. Research Objective

The main goal of the cognitive radar is to estimate the target’s state from the uncertain radar measurements while maintaining low estimation error on a cycle-by-cycle basis. Given the model assumptions, this work aims to demonstrate how the cognitive radar can manipulate the waveform signal parameters to improve the target’s state estimate for the next time instant. In this regard, we mathematically express the design objective in terms of a state estimation problem and propose three research questions on modeling the information processor and the measurement-selection mechanism.

Suppose the motion dynamics of the vehicle-following scenario are expressed by Equation (1); the state estimation problem becomes finding the estimated state, denoted as ${\hat{x}}_{k}∼p\left(x_{k}|Z_{k}\right)$, that minimizes the following objective function at each time step:

(8) $$\underset{{\hat{x}}_{k}\in R^{n}}{\arg\;\min}\;E\left[{\tilde{x}}_{k}^{T}{\tilde{x}}_{k}\right]$$

where ${\tilde{x}}_{k}=x_{k}-{\hat{x}}_{k}$ is the error between the true state and the estimated state, and $p(x_{k}|Z_{k})$ is the probability density function (PDF) of the estimated target’s state. Given that the radar measurements are available up to time *k*, $Z_{k}=\left\{z_{i},i\leq k\right\}$, Equation (8) estimates the target’s state that minimizes the mean squared error. To accomplish this objective, this paper focuses on modeling the information processor and the measurement-selection technique by proposing the following research problems.

**Research Problem** **1.** *Let us assume that the parameters of the model in Equation (1) are known. Determine the amount of new information in radar measurements that contribute to estimating the target’s state ${\hat{x}}_{k}$.*

The first research problem captures the essence of the information processor. Computing the information of the estimated target’s state is crucial because the sensor determines the optimum waveform (or the measurement noise covariance) according to the information processor. Meanwhile, the following research problem deals with how the radar sensor can change to improve the estimate of the target’s state.

**Research Problem** **2.** *Let us assume that the measurement noise covariance, $R_{k}$, can change at any time cycle. Based on Research Problem 1, develop an optimal selection methodology to minimize estimation error and achieve informative measurements with respect to the measurement noise covariance.*

This problem represents the measurement-selection procedure that executes a waveform, leading to a better estimate of the target’s state. It solves an optimization problem that depends on the choice of information measure. Finally, we contemplate a general setting, which combines Research Problem 1 and Research Problem 2 in designing the cognitive radar.

**Research Problem** **3.** *Let us consider that a set of measurement noise covariances $R_{k}^{L}$ are available (i.e., $R_{k}^{L}=\left\{R^{\left(1\right)},R^{\left(2\right)},\cdots ,R^{\left(N_{L}\right)}\right\}$). Derive the algorithm for the information processor and the measurement-selection procedure by looking forward to one time-step ahead. In addition, is it possible to extend the algorithm to acquire informative measurements by planning L steps in advance?*

## 3. Proposed Method

This section briefly reviews the state-of-the-art designs to model the information processor and its corresponding measurement-selection criteria. The paper addresses Haykin’s strategy specific to cognitive radar [27] and, for the first time, discusses alternative approaches that can apply to designing such systems [16,30]. Finally, our proposed solutions to Research Problems 1, 2, and 3 are presented.

### 3.1. Prior Works

Multiple measures are suggested in statistics and information theory to quantify information [14,15,16,30]. The most common is the Shannon entropy, which measures the amount of self-information or (Shannon surprise) of a particular observation, averaged over all possible outcomes [14]. In [27,28], the authors adopt the Shannon entropy to measure the information of the estimated target’s state and model the information processor as follows:

(9) $$H_{k}=-\int_{x_{k}\in R^{n}}p\left(x_{k}|Z_{k}\right)\ln p\left(x_{k}|Z_{k}\right)\;dx_{k}$$

where $p(x_{k}|Z_{k})$ is the posterior PDF of the estimated state. Haykin derives $H_{k}$ in terms of the estimated state covariance, P(k|k), when the Kalman filter is applied for state estimation (i.e., $p\left(x_{k}|Z_{k}\right)=N\left(\hat{x}\left(k|k\right),P\left(k|k\right)\right)$). According to [27,28], the measurement-selection procedure chooses the measurement noise covariance that minimizes the Shannon entropy.

While Haykin considers an information-theoretic approach to design cognitive radar, refs. [15,16] use surprise as the principal mechanism to acquire information. Surprise measures ”how much wow” one experiences when encountering uncertain events [13]. The Bayesian surprise measures the Kullback–Leibler (KL) information between a prior probability distribution and its update when a new observation is made [15]. Based on the research objective, the Bayesian surprise is defined as

(10) $$\begin{array}{c} S_{k}^{B}\left(z_{k}\right)=D_{KL}\left(p\left(x_{k}|Z_{k-1}\right),p\left(x_{k}|Z_{k}\right)\right)=\int_{x_{k}\in R^{n}}p\left(x_{k}|Z_{k-1}\right)\ln\frac{p(x_{k}|Z_{k-1})}{p(x_{k}|Z_{k})}dx_{k} \end{array}$$

where it determines the effect of the new radar measurement $z_{k}$ on the target’s state estimation by measuring the KL distance from the predicted PDF to the posterior PDF. In addition, free energy is another type of surprise that measures the information of a new measurement by weighting and averaging it over all possible models [16]. The free energy is determined as follows:

(11) $$\begin{array}{c} F_{k}\left(z_{k}\right)=-\int_{x_{k}\in R^{n}}p\left(x_{k}|Z_{k-1}\right)\ln p\left(z_{k}|x_{k},Z_{k-1}\right)dx_{k}=S_{k}^{B}\left(z_{k}\right)-\ln p(z_{k}|Z_{k-1}) \end{array}$$

where $p(z_{k}|x_{k},Z_{k-1})$ is the probability of the measurements at time *k*, conditioned on the state and all past measurements. As shown, free energy is expressed in terms of Bayesian surprise and $-\ln p(z_{k}|Z_{k-1})$, which refers to the Shannon surprise within the measurements. Note that the Bayesian surprise and the free energy have been adopted in many works to explain information-seeking behaviors in biological agents [17,19,20,21,22,35,36]. However, these two have not been directly applied to the design of cognitive radar systems.

A classical estimation/control methodology to evaluate the impact of new measurements is computing the trace of the influence matrix that is used in data assimilation for weather forecasting applications [30]. The influence matrix is also suitable for measuring radar measurements’ contribution to estimating the target’s state. For a specific configuration in which the Kalman filter is used for state estimation, the influence matrix is obtained as follows:

(12) $$\begin{array}{c} S_{k}=\frac{\partial H_{k}\hat{x}\left(k|k\right)}{\partial z_{k}}=H_{k}K_{k} \end{array}$$

where $K_{k}$ is the Kalman gain. In [30], the authors determine that the measurement with the maximum trace contributes more information to state estimation. Thus, the measurement noise covariance that maximizes the trace of the influence matrix is selected.

### 3.2. Solution to Research Problem 1

This paper proposes the Bayesian surprise as the main approach to quantifying the amount of new information within the estimated state. The Bayesian surprise demonstrates how the uncertainty in measurements improves future state estimations given prior estimates and provides valuable information to reduce estimation errors over time.

Since the radar measurements are constructed from linear Gaussian state-space models (see Equation (1)), and it is assumed that the parameters of the model are known, the Kalman filter is adopted for state estimation [26]. The Kalman filter is the optimal estimator in the mean square error sense that solves the research objective given in Equation (8). The filter estimates the state mean, $\hat{x}\left(k|k\right)=E\left[x_{k}|Z_{k}\right]$, and its covariance matrix, $P\left(k|k\right)=E[\left(x_{k}-\hat{x}\left(k|k\right)\right){\left(x_{k}-\hat{x}\left(k|k\right)\right)}^{T}|Z_{k}]$, in an iterative manner. Algorithm 1 presents the two-step state prediction and estimation of the Kalman filter.
**Algorithm 1:** Kalman filter [26].**Measurement update (Estimation):**$\hat{x}\left(k|k\right)=\hat{x}\left(k|k-1\right)+K_{k}\left(z_{k}-H_{k}\hat{x}\left(k|k-1\right)\right)$$P\left(k|k\right)=\left(I_{n\times n}-K_{k}H_{k}\right)P\left(k|k-1\right)$$K_{k}=P\left(k|k-1\right)H_{k}^{T}P_{\tilde{z}}{\left(k|k-1\right)}^{-1}$**Time update (Prediction):**$\hat{x}\left(k+1|k\right)=F_{k}\hat{x}\left(k|k\right)$$P\left(k+1|k\right)=Q_{k}+F_{k}P\left(k|k\right)F_{k}^{T}$

Given that $p\left(x_{k}|Z_{k-1}\right)=N\left(\hat{x}\left(k|k-1\right),P\left(k|k-1\right)\right)$ and $p\left(x_{k}|Z_{k}\right)=N\left(\hat{x}\left(k|k\right),P\left(k|k\right)\right)$ are available from the Kalman filter, the following expression for the Bayesian surprise is achieved [37]:

(13) $$\begin{array}{cc} S_{k}^{B}\left(z_{k}\right) & =\frac{1}{2}\left[\ln\frac{\left|P(k|k)\right|}{\left|P(k|k-1)\right|}+\mathrm{tr}\left\{P{\left(k|k\right)}^{-1}P\left(k|k-1\right)\right\}-n+\left|\right|\hat{x}\left(k|k\right)-\hat{x}\left(k|k-1\right){\left|\right|}_{P{\left(k|k\right)}^{-1}}^{2}\right] \end{array}$$

where $\hat{x}\left(k|k-1\right)$, P(k|k−1), and *n* are the predicted state mean, the predicted state covariance matrix, and the state space dimension, respectively. While the above expression demonstrates the Bayesian surprise in state space, the nature of the research problem requires rewriting Equation (13) in measurement space. In this regard, we determine the Bayesian surprise in measurement space as

(14) $$\begin{array}{c} S_{k}^{B}\left(z_{k}\right)=\frac{1}{2}\left[\ln|R_{k}P_{\tilde{z}}{\left(k|k-1\right)}^{-1}\left|-m+|\right|\tilde{z}\left(k|k-1\right){\left|\right|}_{K_{k}^{T}P{\left(k|k\right)}^{-1}K_{k}}^{2}+\mathrm{tr}\left\{{\left(R_{k}P_{\tilde{z}}{\left(k|k-1\right)}^{-1}\right)}^{-1}\right\}\right] \end{array}$$

where $\tilde{z}\left(k|k-1\right)=z_{k}-H_{k}\hat{x}\left(k|k-1\right)$ is the innovation vector, $P_{\tilde{z}}\left(k|k-1\right)=R_{k}+H_{k}P\left(k|k-1\right)H_{k}^{T}$ is the innovation covariance, and *m* is the dimension of the measurement space. Equation (14) clearly shows the connection between the Bayesian surprise and the design parameters of the radar sensor (i.e., $R_{k}$); it also indicates how the information in a current radar measurement influences the Bayesian surprise (i.e., $P_{\tilde{z}}{\left(k|k-1\right)}^{-1}$).

As shown in (14), the Bayesian surprise at time *k* is a function of the measurement $z_{k}$. In a case where $z_{k}$ is not available (e.g., multiple-step planning), this paper proposes computing the expectation of Bayesian surprise instead. The expectation of Bayesian surprise with respect to $p\left(z_{k}|Z_{k-1}\right)∼N\left(H_{k}\hat{x}\left(k|k-1\right),P_{\tilde{z}}\left(k|k-1\right)\right)$ is obtained as follows:

(15) $$\begin{array}{c} E_{p(z_{k}|Z_{k-1})}\left[S_{k}^{B}\left(z_{k}\right)\right]=\frac{1}{2}\ln\left|R_{k}P_{\tilde{z}}{\left(k|k-1\right)}^{-1}\right|+\mathrm{tr}\left\{{\left(R_{k}P_{\tilde{z}}{\left(k|k-1\right)}^{-1}\right)}^{-1}\right\}-m \end{array}$$

where $E_{p(z_{k}|Z_{k-1})}\left[|\right|\tilde{z}{\left(k|k-1\right)||}_{K_{k}^{T}P{\left(k|k\right)}^{-1}K_{k}}^{2}]$ is simplified to $\mathrm{tr}\{{\left(R_{k}P_{\tilde{z}}{\left(k|k-1\right)}^{-1}\right)}^{-1}\}-m$. The measurement noise covariance, $R_{k}$, and the information within the innovation, $P_{\tilde{z}}{\left(k|k-1\right)}^{-1}$, are the only terms that appear in Equation (15). Equation (15) shows that the uncertainty in measurements, balanced by what the filter thinks about the measurements (i.e., $R_{k}P_{\tilde{z}}{\left(k|k-1\right)}^{-1}$), impacts the expectation of Bayesian surprise.

### 3.3. Solution to Research Problem 2

This section explores the measurement-selection scheme associated with the choice of the information processor. Since the Bayesian surprise and its expectation are proposed to solve Research Problem 1, the challenge of Research Problem 2 becomes achieving an optimum selection mechanism based on the Bayesian surprise (or its expectation). To this end, let us first refer to the definition of the influence matrix given in Equation (12). A connection exists between the expected Bayesian surprise and the influence matrix. This relation is evident when rewriting Equation (12) in the measurement space. By expressing the Kalman gain as $K_{k}=P\left(k|k\right)H_{k}^{T}R_{k}^{-1}$ and applying the matrix inversion lemma to Equation (12), the following definition is obtained:

(16) $$\begin{array}{c} S_{k}=I_{m\times m}-R_{k}P_{\tilde{z}}{\left(k|k-1\right)}^{-1} \end{array}$$

where $I_{m\times m}$ is the identity matrix. The term $R_{k}P_{\tilde{z}}{\left(k|k-1\right)}^{-1}$ appears in the expectation of Bayesian surprise as well. The influence matrix is a projection matrix (i.e., $S_{k}$ is symmetric and idempotent); a positive semi-definite matrix and all its diagonal elements are bounded between 0 and 1 [30]. Since the magnitude of the diagonal values of $S_{k}$ corresponds to the influence of the measurement, the trace of the influence matrix is acceptable for determining the impact of measurements [30]. An informative measurement that contributes to state estimation maximizes the trace of the influence matrix.

The influence matrix trace provides insight into how to select informative radar measurements with respect to the Bayesian surprise. According to the properties of the influence matrix, its trace does not exceed the dimension of the measurement space *m*. Therefore, the maximization of $\mathrm{tr}\{S_{k}\}$ is equivalent to minimizing $\mathrm{tr}\{R_{k}P_{\tilde{z}}{\left(k|k-1\right)}^{-1}\}$ (see Equation (16)). In this regard, the measurement-selection procedure concerning the influence matrix becomes solving the following optimization problem:

(17) $$R_{k}^{min}=\underset{R_{k}}{\mathrm{argmin}}\;\mathrm{tr}\left\{R_{k}P_{\tilde{z}}{\left(k|k-1\right)}^{-1}\right\}$$

where $R_{k}^{min}$ is obtained when $\mathrm{tr}\{R_{k}P_{\tilde{z}}{\left(k|k-1\right)}^{-1}\}$ is minimized. To do so, the trace is differentiated with respect to the measurement noise covariance and is set equal to zero:

(18) $$\begin{array}{c} \frac{\partial }{\partial R_{k}}\left(\mathrm{tr}\{R_{k}P_{\tilde{z}}{\left(k|k-1\right)}^{-1}\}\right)=P_{\tilde{z}}{\left(k|k-1\right)}^{-1}=0_{m\times m} \end{array}$$

The following is obtained by substituting the expression for $P_{\tilde{z}}\left(k|k-1\right)$ and employing the matrix inversion lemma:

(19) $$\begin{array}{c} R_{k}^{-1}\left(I_{m\times m}-H_{k}P\left(k|k\right)H_{k}^{T}R_{k}^{-1}\right)=0_{m\times m} \end{array}$$

where $R_{k}^{-1}=0_{m\times m}$ is not applicable; hence, $R_{k}^{min}=H_{k}P\left(k|k\right)H_{k}$. For $R_{k}=H_{k}P\left(k|k\right)H_{k}$, the trace of the influence matrix reaches its maximum.

To demonstrate how the selection criteria for the expectation of Bayesian surprise changes when $R_{k}=H_{k}P\left(k|k\right)H_{k}$, it is suitable to revise $E[S_{k}^{B}\left(z_{k}\right)]$ in terms of the trace operator:

(20) $$\begin{array}{c} E_{p(z_{k}|Z_{k-1})}\left[S_{k}^{B}\left(z_{k}\right)\right]=\frac{1}{2}\mathrm{tr}\left\{\ln\left(R_{k}P_{\tilde{z}}{\left(k|k-1\right)}^{-1}\right)\right\}+\mathrm{tr}\left\{{\left(R_{k}P_{\tilde{z}}{\left(k|k-1\right)}^{-1}\right)}^{-1}-I_{m\times m}\right\} \end{array}$$

where for any positive semi-definite square matrix A (i.e., $R_{k}P_{\tilde{z}}{\left(k|k-1\right)}^{-1}$), ln|A|=tr{ln(A)}. According to the bounds of the natural logarithm, if $A<<I_{m\times m}$, then $\mathrm{abs}\left(\ln A\right)<(A^{-1}-I_{m\times m}$). In other words, the growth rate of $\left(A^{-1}-I_{m\times m}\right)$ is higher than lnA when $A\to 0_{m\times m}$. Applying this condition to Equation (20) for $R_{k}P_{\tilde{z}}{\left(k|k-1\right)}^{-1}\to 0_{m\times m}$ makes it safe to say that $E_{p(z_{k}|Z_{k-1})}\left[S_{k}^{B}\left(z_{k}\right)\right]\approx \mathrm{tr}\left\{{\left(R_{k}P_{\tilde{z}}{\left(k|k-1\right)}^{-1}\right)}^{-1}-I_{m\times m}\right\}$. Therefore, when $R_{k}P_{\tilde{z}}{\left(k|k-1\right)}^{-1}\to 0_{m\times m}$ (or $R_{k}=H_{k}P\left(k|k\right)H_{k}$), the expectation of Bayesian surprise reaches its maximum value. In an informative radar measurement that decreases the state estimation error, the measurement noise covariance is small, and the inverse of the innovation covariance is maximized.

To further elaborate, we consider a simple example by setting $R_{k}P_{\tilde{z}}{\left(k|k-1\right)}^{-1}=\alpha I_{m\times m}$, where 0≤α≤1. We examine two extreme cases of α=0.01 and α=0.99. For α=0.01, the expectation of Bayesian surprise becomes

(21) $$\begin{array}{c} E\left[S_{k}^{B}\left(z_{k}\right)\right]{|}_{\alpha =0.01}=\frac{1}{2}\ln(|0.01I_{m\times m}\left|\right)+\mathrm{tr}\left\{100I_{m\times m}\right\}-m\approx 97m, \end{array}$$

and for α=0.99, $E[S_{k}^{B}\left(z_{k}\right)]$ is determined as

(22) $$\begin{array}{c} E\left[S_{k}^{B}\left(z_{k}\right)\right]{|}_{\alpha =0.99}=\frac{1}{2}\ln(|0.99I_{m\times m}\left|\right)+\mathrm{tr}\left\{{\left(0.99\right)}^{-1}I_{m\times m}\right\}-m\approx 0.005m. \end{array}$$

Evidently, as α→0, the expectation of Bayesian surprise reaches a higher value than the case where α→1. Moreover, the final two terms in Equation (21) (i.e., $\mathrm{tr}\{100I_{m\times m}\}-m=99$*m* ) are dominant as α approaches zero. Hence, when $R_{k}P_{\tilde{z}}{\left(k|k-1\right)}^{-1}\to 0_{m\times m}$, the expectation of Bayesian surprise is maximized.

### 3.4. Solution to Research Problem 3

The solution to the final research problem aligns with the discussions carried out in the last two sections. As assumed, the sensor profile library, depicted in Figure 1b, withholds a set of measurement noise covariances, defined as $R_{k}^{L}=\left\{R^{\left(1\right)},R^{\left(2\right)},\cdots ,R^{\left(N_{L}\right)}\right\}$. At time cycle *k*, the cognitive radar estimates the target’s state from the radar measurements. Through multiple stages of prediction and estimation (i.e., planning), the information processor measures the contribution of each measurement noise covariance to estimating the target’s future state based on the expectation of Bayesian surprise. Eventually, the radar selects the measurement noise covariance with the maximum expectation of Bayesian surprise.

Given that the estimated state covariance, P(k|k), is accessible from the state estimation process, the proposed algorithm for one-step planning is summarized in Algorithm 2. Algorithm 2 presents the step-by-step procedure for obtaining the expected Bayesian surprise values corresponding to the *i*-th measurement noise covariance, $i=1,\cdots ,N_{L}$. To this end, the one-step measurement-selection mechanism based on the expectation of Bayesian surprise is demonstrated as follows:

(23) $$i^{☆}=\underset{i=\{1,\cdots ,N_{L}\}}{\mathrm{argmax}}\;E\left[S_{k+1}^{B^{\left(i\right)}}\right]$$

where $R_{k+1}=R^{\left(i^{☆}\right)}\in R_{k}^{L}$ leads to a better estimate of the target’s state for time k+1. The waveform associated with $R_{k+1}$ is applied to the radar environment and sets a repeat of the cycle.
**Algorithm 2:** Cognitive radar for one-step planning.1: P(k|k) and $R_{k}^{L}=\left\{R^{\left(1\right)},R^{\left(2\right)},\cdots ,R^{\left(N_{L}\right)}\right\}$ are available at time *k*2: $P\left(k+1|k\right)=Q_{k}+F_{k}P\left(k|k\right)F_{k}^{T}$3: **for $i=1,\cdots ,N_{L}$**4: $\;P_{\tilde{z}}^{\left(i\right)}\left(k+1|k\right)=R_{k+1}^{\left(i\right)}+H_{k+1}P\left(k+1|k\right)H_{k+1}^{T}$5: Compute $E[S_{k+1}^{B^{\left(i\right)}}]$ from (15)6: **end for**7: $i^{☆}=\underset{i}{\mathrm{argmax}}\;E\left[S_{k+1}^{B^{\left(i\right)}}\right]$ for $i=1,\cdots ,N_{L}$8: $R_{k+1}=R^{\left(i^{☆}\right)}\in R_{k}^{L}$

Table 1 summarizes alternative models of the information processor and its corresponding measurement-selection procedure for one-step planning. The expressions are derived when the Kalman filter is presumed for state estimation. It is straightforward to follow Haykin’s design [27,28] and the influence matrix approach [30]. However, a detailed description of solving the research problems with respect to free energy is carried out in Appendix A. The authors make a similar case for using the expectation of free energy to model the information processor instead of the free energy itself. In addition, the measurement noise covariance that maximizes the expectation of free energy minimizes the state estimation error for the upcoming cycle. Haykin’s method requires an additional step to calculate P(k+1|k+1), while the other three share the same term and exclude this extra step. Although Haykin’s design implies the connection to the measurement noise covariance, his approach is aligned with the research objective. This is because it reduces the estimation error of the target’s state by minimizing the estimated state covariance, P(k+1|k+1).

This paper also presents the means that extend the one-step planning algorithm to *L* steps. Since P(k|k) and the entire sensor profile library are available, the Kalman algorithm is partially applicable to predict the state covariance and to compute the innovation covariance and the estimated state covariance. The only difference is that the Kalman algorithm repeats *L* times to capture the influence of *L* future measurements. In this regard, the expectation of Bayesian surprise at time k+L is calculated as follows:

(24) $$\begin{array}{c} E\left[S_{k+L}^{B^{\left(ij\cdots l\right)}}\right]=\frac{1}{2}\ln\left|R_{k+L}^{\left(l\right)}P_{\tilde{z}}^{\left(ij\cdots l\right)}{\left(k+L|k+L-1\right)}^{-1}\right|+\mathrm{tr}\left\{{\left(R_{k+L}^{\left(l\right)}P_{\tilde{z}}^{\left(ij\cdots l\right)}{\left(k+L|k+L-1\right)}^{-1}\right)}^{-1}\right\}-m \end{array}$$

where (ij⋯l) represents the *L*-length sequence of measurement noise covariances. Note that the expectation is computed with respect to $p(z_{k+L}|Z_{k+L-1})$, with mean and covariance, $H_{k+L}{\hat{x}}^{\left(ij\cdots l\right)}\left(k+L|k+L-1\right)$ and $P_{\tilde{z}}^{\left(ij\cdots l\right)}\left(k+L|k+L-1\right)$, respectively. Consequently, the sequence with the maximum expectation of Bayesian surprise leads to informative measurements:

(25) $$\left(i^{☆}j^{☆}\cdots l^{☆}\right)=\underset{i,j,\cdots ,l=\{1,\cdots ,N_{L}\}}{\mathrm{argmax}}\;E\left[S_{k+L}^{B^{\left(ij\cdots l\right)}}\right]$$

where $R_{k+1}=R^{\left(i^{☆}\right)}\in R_{k}^{L}$. Algorithm 3 illustrates the proposed cognitive radar algorithm for *L*-step planning. Note that a similar process applies to the other models for *L*-step planning with some minor modifications.
**Algorithm 3:** Cognitive radar for *L*-step planning.1: P(k|k) and $R_{k}^{L}=\left\{R^{\left(1\right)},R^{\left(2\right)},\cdots ,R^{\left(N_{L}\right)}\right\}$ are available at time *k*2: $P\left(k+1|k\right)=Q_{k}+F_{k}P\left(k|k\right)F_{k}^{T}$3: **for $i=1,\cdots ,N_{L}$**4: $\;P_{\tilde{z}}^{\left(i\right)}\left(k+1|k\right)=R_{k+1}^{\left(i\right)}+H_{k+1}P\left(k+1|k\right)H_{k+1}^{T}$5: $\;P^{\left(i\right)}\left(k+1|k+1\right)=P\left(k+1|k\right)-P\left(k+1|k\right)H_{k+1}^{T}P_{\tilde{z}}^{\left(i\right)}{\left(k+1|k\right)}^{-1}H_{k+1}P\left(k+1|k\right)$6: **for $j=1,\cdots ,N_{L}$**7: $\;P^{\left(i\right)}\left(k+2|k+1\right)=F_{k+1}P^{\left(i\right)}\left(k+1|k+1\right)F_{k+1}^{T}+Q_{k+1}$8: $\;P_{\tilde{z}}^{\left(ij\right)}\left(k+2|k+1\right)=R_{k+2}^{\left(j\right)}+H_{k+2}P^{\left(i\right)}\left(k+2|k+1\right)H_{k+2}^{T}$9: $\;P^{\left(ij\right)}\left(k+2|k+2\right)=P^{\left(i\right)}\left(k+2|k+1\right)\left[I_{n\times n}-H_{k+2}^{T}P_{\tilde{z}}^{\left(ij\right)}{\left(k+2|k+1\right)}^{-1}H_{k+2}P^{\left(i\right)}\left(k+2|k+1\right)\right]$10: ⋯11:  **for $l=1,\cdots ,N_{L}$**12:  $\;P^{\left(ij\cdots r\right)}\left(k+L|k+L-1\right)=F_{k+L-1}P^{\left(ij\cdots r\right)}\left(k+L|k+L-1\right)F_{k+L-1}^{T}+Q_{k+L-1}$13:  $\;P_{\tilde{z}}^{\left(ij\cdots rl\right)}\left(k+L|k+L-1\right)=R_{k+L}^{\left(l\right)}+H_{k+L}P^{\left(ij\cdots r\right)}\left(k+L|k+L-1\right)H_{k+L}^{T}$14:  Compute $E[S_{k+L}^{B^{\left(ij\cdots rl\right)}}]$ from (24)15: **end for**16: ⋯17: **end for**18: **end for**19: $\left(i^{☆}j^{☆}\cdots r^{☆}l^{☆}\right)=\underset{i}{\mathrm{argmax}}\;E\left[S_{k+L}^{B^{\left(ij\cdots rl\right)}}\right]$ for $i,j,\cdots ,r,l=1,\cdots ,N_{L}$20: $R_{k+1}=R^{\left(i^{☆}\right)}\in R_{k}^{L}$

## 4. Numerical Results

In this section, simulation results are presented to compare the state estimation performance of the proposed cognitive radar with the state-of-the-art listed in Table 1. The following demonstrates the experimental setup and parameter settings for generating radar measurements that emulate the simple vehicle-following scenario in Figure 1a. The paper suggests a radar configuration suitable for single-target tracking in highway and urban driving environments. Several error metrics are introduced to examine various aspects of the estimation performance. This section compares the system performance of the proposed one-step planning algorithm to its alternative competitors for different state estimation errors through a series of experiments. In addition, this section also analyzes the impact of multiple-step planning in improving state estimation performance. Results are verified over numerous Monte Carlo runs.

### 4.1. Simulation Setup and Data Generation

The purpose of the experiment is to evaluate the estimation performance of the proposed cognitive radar. Since the paper adopts the Kalman filter to accomplish state estimation, the model parameters in Equation (1) (i.e., $F_{k}$, $Q_{k}$, $H_{k}$, $R_{k}$, $\hat{x}\left(0|0\right)$ and P(0|0)) are assumed available. In this regard, the radar sensor configuration and the parameter setting for generating radar measurements are presented.

**Figure 2:**
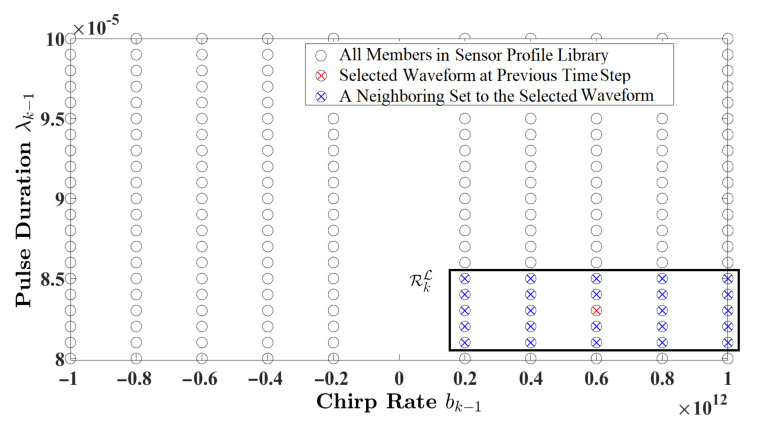
An example of a neighboring set $R_{k}^{L}$ with 25 members.

For the vehicle-following scenario depicted in Figure 1a, the simulation assumes that the two cars are moving forward in the same lane (i.e., $d_{y}=0$). In this simulation, the FMCW radar sensor is mounted on the host vehicle and operates in the 77 GHz frequency band for short- and long-range applications [31]. The bandwidth of the transmitted radar signal is set to B=100 MHz, and 0 dB SNR is achieved at $d_{0}=2000$ m. According to Equation (6), the measurement noise covariance depends on the choice of pulse duration and the chirp rate, $R_{k}\left(\lambda_{k-1},b_{k-1}\right)$. By assuming that the radar sensor maintains a maximum range of $d_{max}=100$ m and a maximum velocity of $v_{max}=100$ m/s, the sensor profile library consists of measurement noise covariances specified for the following values:

$$\begin{array}{cc} \; & \lambda_{k-1}\in \left[1\times 10^{-6}:10^{-7}:10\times 10^{-6}\right]\cup \left[1.1\times 10^{-5}:10^{-6}:10\times 10^{-5}\right],\\ & b_{k-1}\in \left[-1\times 10^{12}:0.2\times 10^{12}:-0.2\times 10^{12}\right]\cup \left[0.2\times 10^{12}:0.2\times 10^{12}:1\times 10^{12}\right]. \end{array}$$

where $\lambda_{k-1}$ and $b_{k-1}$ are configured to simulate a practical radar sensor for single-target tracking applications [6]. The sensor profile library is composed of N=1810 measurement noise covariances, denoted as $R={\left\{R^{\left(i\right)}\left(\lambda_{k-1},b_{k-1}\right)\right\}}_{i=1}^{N}$. Since *N* is a large number, and going through the entire library at each time instant is cost-ineffective, this paper adopts the kNN method to obtain a smaller set with $N_{L}=25$ members. Figure 2 illustrates an example of a localized set of measurement noise covariance, $R_{k}^{L}$, that is distinguished by pulse duration and chirp rate.

This article demonstrates highway and urban driving to examine the state estimation performance of the proposed cognitive radar for a realistic vehicle-following scenario. Since the true initial state, $x_{0}$, and its estimation elements (i.e., $\hat{x}\left(0|0\right)$, P(0|0)) depend on the driving environment, without loss of generality, the true initial state for highway driving is set to

$$x_{0}={\left[25\;m/s,3\;{m/s}^{2},100\;m,23\;m/s,2\;{m/s}^{2}\right]}^{T},$$

while the initial estimation of the state mean and its covariance matrix are assumed as

$$\hat{x}\left(0|0\right)={\left[24\;m/s,3\;{m/s}^{2},80\;m,23\;m/s,2\;{m/s}^{2}\right]}^{T},$$

P(0|0)=diag([100,1,100,100,1]).

In the meantime, for the urban driving scenario $x_{0}$, $\hat{x}\left(0|0\right)$, and P(0|0) are set to

$$\begin{array}{cc} \; & x_{0}={\left[13\;m/s,1\;{m/s}^{2},30\;m,12\;m/s,1\;{m/s}^{2}\right]}^{T},\\ \; & \hat{x}\left(0|0\right)={\left[12.5\;m/s,1\;{m/s}^{2},28\;m,12\;m/s,1\;{m/s}^{2}\right]}^{T},\\ \; & P(0|0)=\mathrm{diag}([100,1,100,100,1]). \end{array}$$

where the values are adjusted according to an in-city driving experience. Note that the estimated initial state ${\hat{x}}_{0}∼N\left(\hat{x}\left(0|0\right),P\left(0|0\right)\right)$ is a random value that changes per Monte Carlo run. This simulation sets the state noise variance to $\sigma_{q}^{2}=0.01$ and the sample time to $T_{s}=0.1$ s for computing $F_{k}$ and $Q_{k}$ to ensure constant acceleration.

### 4.2. Evaluation Metrics

This paper considers numerous error measures to evaluate and compare the estimation performance of the proposed cognitive radar to the alternative design in Table 1. The error measures include the root mean square relative error (RMSRE), the average Euclidean relative error (ARE), the harmonic average relative error (HRE), and the geometric average relative error (GRE) [38]. Due to numerical reasons, the logarithm of the GRE is calculated instead, log(GRE). Table 2 provides the mathematical expressions of these error measures. In Table 2, ${\tilde{x}}_{k}^{j}={\hat{x}}^{j}\left(k|k\right)-x_{k}^{j}$, where $x_{k}^{j}$, ${\hat{x}}^{j}\left(k|k\right)$ and ${\tilde{x}}_{k}^{j}$ are, respectively, the state vector, the estimated state vector, and the state estimation error of the *j*-th Monte Carlo simulation at time step *k*. $N_{mc}$ represents the number of Monte Carlo simulations. The paper considers relative error measures since they are suitable for the performance evaluation of an estimation algorithm. However, the absolute value of the error metrics mentioned above—which computes the time average—is justified for ranking the overall state estimation performance of the cognitive radar. The absolute error counterparts of the relative error measures are given in Table 2.

### 4.3. Performance Evaluation and Comparison for One-Step Planning

This section demonstrates the estimation response of the proposed radar design by tracking the velocity of the target vehicle, $v_{x,k}^{1}$, and the longitude distance, $d_{x,k}$, when one-step planning is involved. The experiment examines target tracking in both highway and urban driving. Results are obtained for $N_{mc}=10,000$ Monte Carlo runs. Multiple attributes are considered for ranking the radar designs’ overall estimation performance, as listed in Table 1. This paper applies a pairwise comparison technique that adopts a ranking vector (RV) to compare different estimation algorithms [32]. This method exploits comparison information based on the probability of the relative closeness of competing estimators to the true quantity. The authors in [32] discuss a variety of approaches for determining a unique RV. Here, order-preserving mapping is considered to obtain the RV for ranking state estimation performance. Since this method is straightforward, the paper solely refers to the results of applying this strategy. In addition, the authors of this paper decided only to present the RMSRE curves of the estimation performance to avoid unnecessary repetition. However, the absolute relative error measures (i.e., ARMSRE, AARE, AHRE, and AGRE) are recorded to evaluate the entire estimation performance.

**Figure 3:**
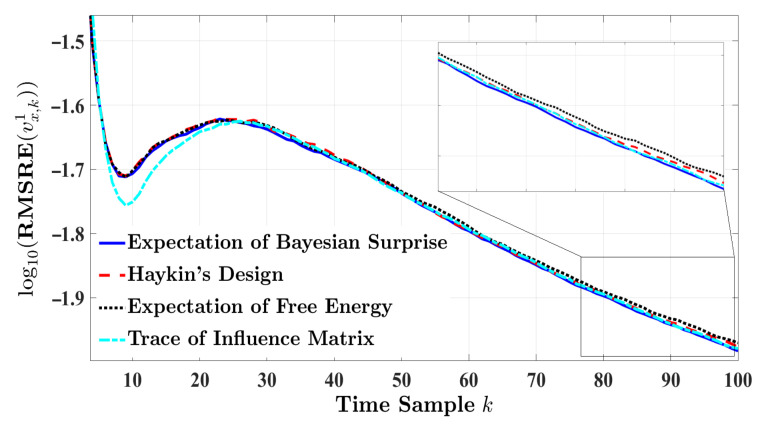
The RMSRE of the target’s velocity for one-step planning in highway driving.

**Figure 4:**
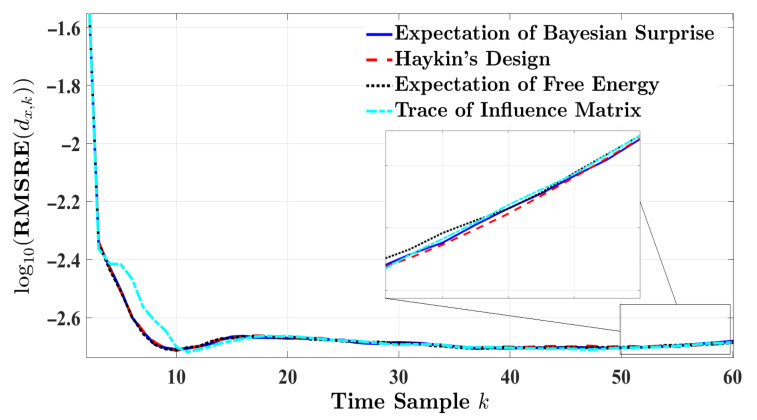
The RMSRE of the longitude distance for one-step planning in highway driving.

Figure 3 and Figure 4, respectively, illustrate the RMSRE performance of velocity and longitude distance of the target vehicle for a highway driving experience. The duration of the experiment is set to 10 s. Although the RMSRE results are plotted in a logarithm scale, the figures show that the estimation response of the four radar models are in close proximity. In this regard, the estimation performance is ranked based on a pairwise comparison method for the mentioned error measures. Table 3 provides the absolute error measure values regarding the velocity of the target vehicle. For the results in Table 3, the RV based on order-preserving mapping is computed as follows:

$$r_{1}={\left[\begin{array}{cccc} 0.8086 & 0.5401 & 0.1650 & 0.1650 \end{array}\right]}^{T},$$

where the order of the elements in $r_{1}$ is similar to the order shown in Table 3. The magnitude reflects the goodness of the approach relative to each other. The larger the value, the better the corresponding estimation performance. According to $r_{1}$, the rank for the velocity of the target vehicle in the highway scenario is $E\left[S_{k+1}^{B}\right]>\mathrm{tr}\left\{S_{k+1}\right\}>E\left[F_{k+1}\right]=H_{k+1}$, indicating that the expectation of Bayesian surprise exceeds the alternative designs of the information processor. $r_{1}$ also implies that the expectation of free energy and Haykin’s Shannon entropy rank similarly in estimation performance.

Furthermore, Table 4 reports the absolute error measures of the longitude distance for highway driving. Apparently, the four radar designs present identical outcomes for 10,000 runs of Monte Carlo simulations. This eventually leads to the following RV:

$$r_{2}={\left[\begin{array}{cccc} 0.5 & 0.5 & 0.5 & 0.5 \end{array}\right]}^{T},$$

where it indicates that the estimation performance of the longitude distance ranks the same for all the radar models.

**Figure 5:**
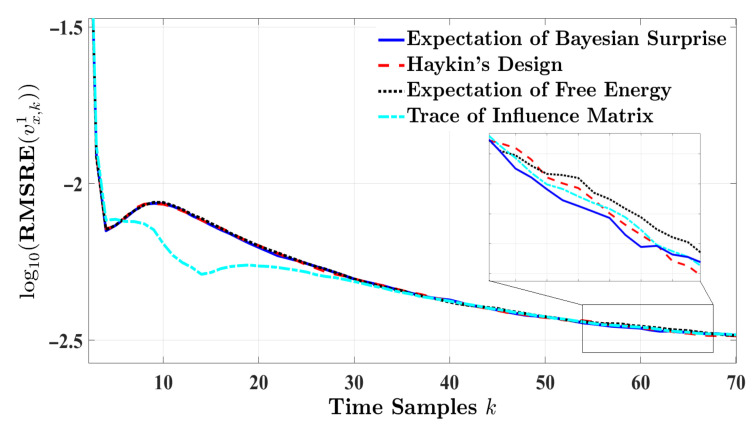
The RMSRE of the target’s velocity for one-step planning in urban driving.

**Figure 6:**
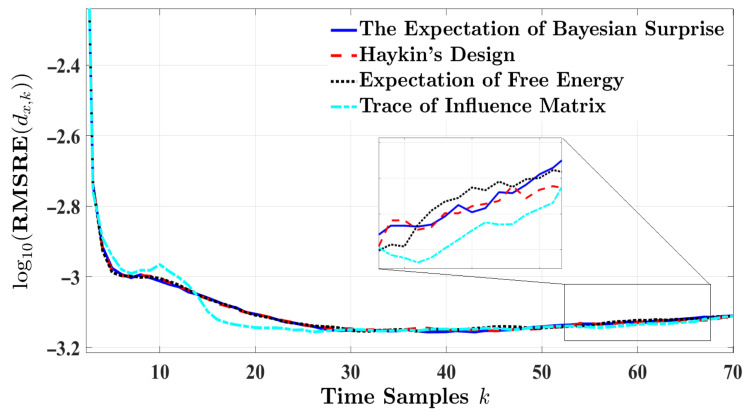
The RMSRE of the longitude distance for one-step planning in urban driving.

Figure 5 and Figure 6, respectively, depict the RMSRE curves of the velocity and longitude distance when the vehicle-following scenario takes place in an urban environment. In this experiment, results are simulated for 7 s. While the RMSRE curves regarding the four designs converge over time, the estimation response based on the trace of the influence matrix experiences the lowest error at earlier time instances. Table 5 displays the different approaches to modeling cognitive radar versus the absolute relative error metrics for estimating the target’s velocity. As expected, the trace of the influence matrix presents a minimum level of error compared to the alternative designs. As a result, the following RV is achieved:

$$r_{3}={\left[\begin{array}{cccc} 0.4926 & 0.7896 & 0.2587 & 0.2587 \end{array}\right]}^{T},$$

where the influence matrix trace ranks the topmost in estimating the velocity. Our proposed scheme is second on the ranking scale. The expectation of Bayesian surprise experiences a more significant error than the trace of the influence matrix, with AHRE as the only exception. The expectation of free energy and Haykin’s design present the poorest estimation. Table 6 provides the overall performance for longitude distance in urban driving. Table 6 shows that all models offer the same outcome for each error measure. Therefore, all designs are ranked equally regarding estimation performance, similar to highway driving.

According to this experiment, the following remarks can be made for one-step planning. In the case of highway driving, the expectation of Bayesian surprise outperforms the other three techniques in estimating the target’s velocity. In the meantime, the trace of influence matrix is a better choice for modeling the information processor in an urban environment. Note that both methods only consider $R_{k+1}P_{\tilde{z}}{\left(k+1|k\right)}^{-1}$ as the means to minimize the state estimation error for the next time instant. This implies that the uncertainty in the measurements balanced by the certainty in innovation provides sufficient information to predict and estimate the target’s dynamic state ahead of time.

### 4.4. Performance Evaluation and Comparison for *L*-Step Planning

**Figure 7:**
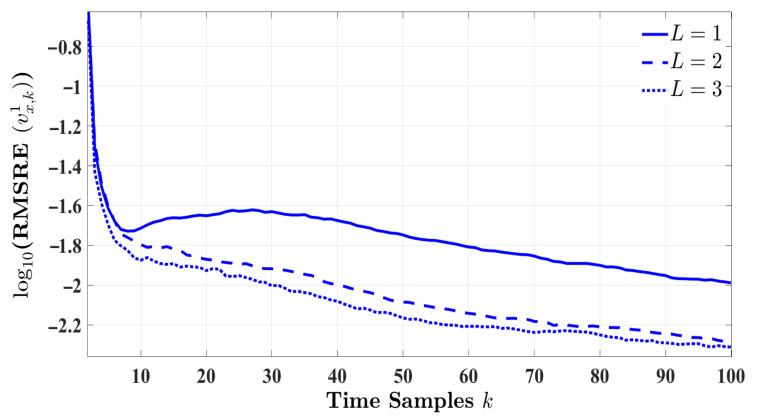
The RMSRE of the target’s velocity for L={1,2,3} and $N_{mc}=1000$ in highway driving.

This experiment evaluates the estimation performance of the proposed cognitive radar when the impact of *L* future measurements are considered in estimating the target’s state for the upcoming time cycle. The results of this experiment are averaged over $N_{mc}=1000$ Monte Carlo simulations for the highway driving scenario. This section examines how $v_{x,k}^{1}$ estimation improves when multiple-step planning is assumed. According to the figures and tables in the previous section, the longitude distance seems invariant for the various error measures. To this end, this experiment focuses only on estimating the target’s velocity. Figure 7 illustrates the RMSRE performance of the proposed radar design for L={1,2,3}. Figure 7 shows that the estimation error is substantially decreased by increasing the planning step from one to two. Although three-step planning outperforms them all, the amount of errors reduced by changing L=2 to L=3 is negligible compared to L=1 to L=2. Additionally, increasing *L* is associated with a longer simulation run time and higher computational complexity. Thus, two-step planning seems the optimum fit to enhance the state estimation performance of the proposed cognitive radar.

**Figure 8:**
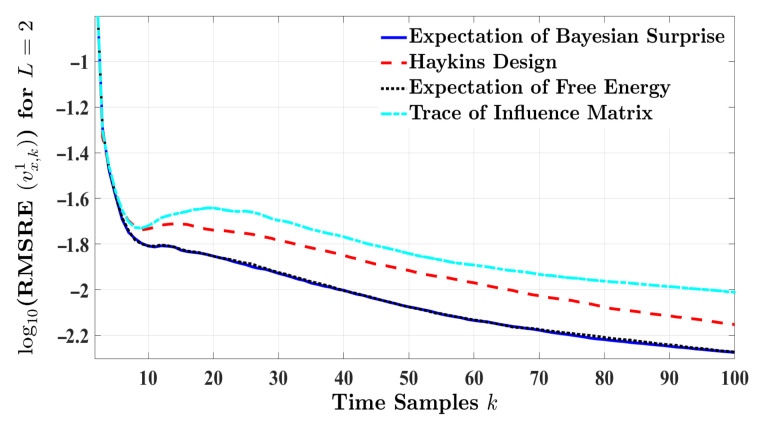
The RMSRE of the target’s velocity for L=2 and $N_{mc}=10,000$ in highway driving.

For two-step planning, this section also analyzes the estimation performance of the proposed cognitive radar with alternative designs. Figure 8 compares the RMSRE curves of the four radar designs by setting L=2 and $N_{mc}=10,000$ for highway driving. According to Figure 8, the expectation of Bayesian surprise and the expectation of free energy present minimum estimation errors with respect to RMSRE, while surpassing the other two techniques. Table 7 supports this claim in terms of the absolute RMSRE. The results indicate that, on average, the expectation of Bayesian surprise improves the estimation process when multiple-step planning is considered.

## 5. Conclusions

This paper proposed a novel design of cognitive radar that plans the estimation response of the system based on the expectation of Bayesian surprise and makes a decision by reducing the estimation error over time. In this work, the radar measurements were expressed for a set of linear Gaussian state-space models to describe the motion dynamics of a simple vehicle-following scenario. Assuming that the model parameters are somehow known, the Kalman filter was applied for state estimation. According to the filter’s estimate, the radar measures how much information each waveform—available from the sensor profile library—contributes to estimating the target’s future state (i.e., velocity, distance), and chooses the one that maximizes the expectation of Bayesian surprise. This research showed that maximizing the expectation of Bayesian surprise leads to informative measurements and successively decreases the state estimation error. In addition, estimation algorithms for one-step planning and multiple-step planning are determined. The paper also demonstrated a unified framework to re-introduce and relate different design methodologies to model cognitive radar systems. Several experiments were carried out to evaluate and compare the estimation performance of the proposed method to alternative designs. Numerical results were implemented to emulate real-life highway and urban driving experiences. The paper examined the credibility of the proposed approach based on a pairwise comparison method for various error measures. Results indicated that the balance between uncertainty in the measurements and the certainty in innovations provides sufficient information for accurate target tracking for one-step planning. The paper also demonstrated that two-step planning improves the estimation error significantly compared to one-step planning. Meanwhile, the proposed radar design exceeds its competitors’ overall estimation performance when two-step planning is applied.

## Figures and Tables

**Table 1:** Information processor and measurement-selection designs for one-step planning.

Method	Information Processor	Measurement Selection
Haykin’s Approach	$H_{k+1}^{\left(i\right)}=\frac{1}{2}\left[\ln|P^{\left(i\right)}{\left(k+1|k+1\right)|+\ln\left(2\pi e\right)}^{n}\right]$	$i^{☆}=\underset{i=\{1,\cdots ,N_{L}\}}{\mathrm{argmin}}\;H_{k+1}^{\left(i\right)}$
Expectation of Bayesian Surprise	$E\left[S_{k+1}^{B^{\left(i\right)}}\right]=\frac{1}{2}\ln\left|R_{k+1}^{\left(i\right)}P_{\tilde{z}}^{\left(i\right)}{\left(k+1|k\right)}^{-1}\right|+\mathrm{tr}\left\{{\left(R_{k+1}^{\left(i\right)}P_{\tilde{z}}^{\left(i\right)}{\left(k+1|k\right)}^{-1}\right)}^{-1}\right\}-m$	$i^{☆}=\underset{i=\{1,\cdots ,N_{L}\}}{\mathrm{argmax}}\;E\left[S_{k+1}^{B^{\left(i\right)}}\right]$
Expectation of Free Energy	$E\left[F_{k+1}^{\left(i\right)}\right]=E\left[S_{k+1}^{B^{\left(i\right)}}\right]+\frac{1}{2}\left[\ln\right|P_{\tilde{z}}^{\left(i\right)}\left(k+1|k\right)\left|+\ln{\left(2\pi e\right)}^{m}\right]$	$i^{☆}=\underset{i=\{1,\cdots ,N_{L}\}}{\mathrm{argmax}}\;E\left[F_{k+1}^{\left(i\right)}\right]$
Trace of Influence Matrix	$\mathrm{tr}\left\{S_{k+1}^{\left(i\right)}\right\}=m-\mathrm{tr}\left\{R_{k+1}^{\left(i\right)}P_{\tilde{z}}^{\left(i\right)}{\left(k+1|k\right)}^{-1}\right\}$	$i^{☆}=\underset{i=\{1,\cdots ,N_{L}\}}{\mathrm{argmax}}\;\mathrm{tr}\left\{S_{k+1}^{\left(i\right)}\right\}$

**Table 2:** Relative error measures and absolute values for performance evaluation [38].

	Relative Error Metric	Absolute of Error Metric
Root mean square relative error (RMSRE)	${\mathrm{RMSRE}}_{k}=\frac{{\left(\sum_{j=1}^{N_{mc}}\left|\right|{\tilde{x}}_{k}^{j}{\left|\right|}^{2}\right)}^{\frac{1}{2}}}{{\left(\sum_{j=1}^{N_{mc}}\left|\right|x_{k}^{j}{\left|\right|}^{2}\right)}^{\frac{1}{2}}}$	$\mathrm{ARMSRE}=\frac{1}{K}\sum_{k=1}^{K}{\mathrm{RMSRE}}_{k}$
Average Euclidean relative error (ARE)	${\mathrm{ARE}}_{k}=\frac{\sum_{j=1}^{N_{mc}}\left|\right|{\tilde{x}}_{k}^{j}\left|\right|}{\sum_{j=1}^{N_{mc}}\left|\right|x_{k}^{j}\left|\right|}$	$\mathrm{AARE}=\frac{1}{K}\sum_{k=1}^{K}{\mathrm{ARE}}_{k}$
Harmonic average relative error (HRE)	${\mathrm{HRE}}_{k}=\frac{{\left(\sum_{j=1}^{N_{mc}}\left|\right|{\tilde{x}}_{k}^{j}{\left|\right|}^{-1}\right)}^{-1}}{{\left(\sum_{j=1}^{N_{mc}}\left|\right|x_{k}^{j}{\left|\right|}^{-1}\right)}^{-1}}$	$\mathrm{AHRE}=\frac{1}{K}\sum_{k=1}^{K}{\mathrm{HRE}}_{k}$
Geometric average relative error (GRE)	$\log\left({\mathrm{GRE}}_{k}\right)=\frac{1}{N_{mc}}\sum_{j=1}^{N_{mc}}\log\frac{\left|\right|{\tilde{x}}_{k}^{j}\left|\right|}{\left|\right|x_{k}^{j}\left|\right|}$	$\log\left(\mathrm{AGRE}\right)=\frac{1}{K}\sum_{k=1}^{K}\log\left({\mathrm{GRE}}_{k}\right)$

**Table 3:** Performance comparison of radar designs versus multiple error measures for estimating the target’s velocity in highway driving.

	$E[S_{k+1}^{B}]$	$\mathrm{tr}\{S_{k+1}\}$	$E[F_{k+1}]$	$H_{k+1}$
ARMSRE	0.0202	0.0203	0.0204	0.0204
AARE	0.0162	0.0162	0.0163	0.0163
AHRE	0.0022	0.0023	0.0023	0.0023
log(AGRE)	−4.68	−4.68	−4.67	−4.67

**Table 4:** Performance comparison of radar designs versus multiple error measures for estimating the longitude distance in highway driving.

	$E[S_{k+1}^{B}]$	$\mathrm{tr}\{S_{k+1}\}$	$E[F_{k+1}]$	$H_{k+1}$
ARMSRE	0.0028	0.0028	0.0028	0.0028
AARE	0.0022	0.0022	0.0022	0.0022
AHRE	$3\times 10^{-4}$	$3\times 10^{-4}$	$3\times 10^{-4}$	$3\times 10^{-4}$
log(AGRE)	−6.67	−6.67	−6.67	−6.67

**Table 5:** Performance comparison of radar designs versus multiple error measures for estimating the target’s velocity in urban driving.

	$E[S_{k+1}^{B}]$	$\mathrm{tr}\{S_{k+1}\}$	$E[F_{k+1}]$	$H_{k+1}$
ARMSRE	0.0081	0.0079	0.0083	0.0083
AARE	0.0059	0.0057	0.006	0.006
AHRE	0.001	0.001	0.001	0.001
log(AGRE)	−5.95	−5.98	−5.92	−5.92

**Table 6:** Performance comparison of radar designs versus multiple error measures for estimating the longitude distance in urban driving.

	$E[S_{k+1}^{B}]$	$\mathrm{tr}\{S_{k+1}\}$	$E[F_{k+1}]$	$H_{k+1}$
ARMSRE	0.0014	0.0014	0.0014	0.0014
AARE	0.001	0.001	0.001	0.001
AHRE	$1\times 10^{-4}$	$1\times 10^{-4}$	$1\times 10^{-4}$	$1\times 10^{-4}$
log(AGRE)	−7.74	−7.74	−7.74	−7.74

**Table 7:** Performance comparison with respect to the ARMSRE of the target’s velocity for two-step planning in highway driving.

	$E[S_{k+1}^{B}]$	$\mathrm{tr}\{S_{k+1}\}$	$E[F_{k+1}]$	$H_{k+1}$
ARMSRE	0.0126	0.0182	0.0127	0.0156

## Appendix A

To model the information processor based on the free energy, let us refer to Equation (11). By replacing $p\left(z_{k}|Z_{k-1}\right)∼N\left(H_{k}\hat{x}\left(k|k-1\right),P_{\tilde{z}}\left(k|k-1\right)\right)$ in Equation (11), the following is achieved:

(A1) $$\begin{array}{c} F_{k}\left(z_{k}\right)=S_{k}^{B}\left(z_{k}\right)+\frac{1}{2}\left[\ln{\left(2\pi \right)}^{m}+\ln|P_{\tilde{z}}\left(k|k-1\right)|+||\tilde{z}\left(k|k-1\right){\left|\right|}_{P_{\tilde{z}}{\left(k|k-1\right)}^{-1}}^{2}\right] \end{array}$$

where $S_{k}^{B}\left(z_{k}\right)$ is given in Equation (14). Similar to the Bayesian surprise, free energy is a function of $z_{k}$. In the case that $z_{k}$ is unavailable, the expectation of the free energy with respect to $p(z_{k}|Z_{k-1})$ is obtained instead:

(A2) $$\begin{array}{c} E_{p(z_{k}|Z_{k-1})}\left[F_{k}\left(z_{k}\right)\right]=E\left[S_{k}^{B}\left(z_{k}\right)\right]+\frac{1}{2}\left[\ln|P_{\tilde{z}}{\left(k|k-1\right)|+\ln\left(2\pi e\right)}^{m}\right] \end{array}$$

where the second term is the expectation of $-\ln p(z_{k}|Z_{k-1})$. Compared to the expectation of Bayesian surprise, Equation (A2) captures the filter’s uncertainty in interpreting the radar measurements. A recent study adopts the expectation of free energy as a means to investigate exploratory behavior in linear Gaussian dynamic systems [39].

An equivalent analysis of the measurement-selection procedure based on the expectation of Bayesian surprise also applies to the expectation of free energy. With some basic manipulations of Equation (A2), the expectation of free energy reaches its maximum when $R_{k}P_{\tilde{z}}{\left(k|k-1\right)}^{-1}\to 0_{m\times m}$ (or $R_{k}=H_{k}P\left(k|k\right)H_{k}$). In this regard, the measurement noise covariance that maximizes the expectation of free energy leads to a better estimate of the target’s state at the succeeding time instant.
